# Astrocytes in Chronic Pain: Cellular and Molecular Mechanisms

**DOI:** 10.1007/s12264-022-00961-3

**Published:** 2022-11-14

**Authors:** Huan-Jun Lu, Yong-Jing Gao

**Affiliations:** Institute of Pain Medicine and Special Environmental Medicine, Co-innovation Center of Neuroregeneration, Nantong University, Nantong, 226019 China

**Keywords:** Astrocyte, Microglia, Neuron-glia interaction, Spinal cord, Chronic pain

## Abstract

Chronic pain is challenging to treat due to the limited therapeutic options and adverse side-effects of therapies. Astrocytes are the most abundant glial cells in the central nervous system and play important roles in different pathological conditions, including chronic pain. Astrocytes regulate nociceptive synaptic transmission and network function *via* neuron–glia and glia–glia interactions to exaggerate pain signals under chronic pain conditions. It is also becoming clear that astrocytes play active roles in brain regions important for the emotional and memory-related aspects of chronic pain. Therefore, this review presents our current understanding of the roles of astrocytes in chronic pain, how they regulate nociceptive responses, and their cellular and molecular mechanisms of action.

## Introduction

Pain, as defined by the International Association for the Study of Pain, is an unpleasant sensory and emotional experience associated with, or resembling that associated with actual or potential tissue damage. Under physiological conditions, pain plays a protective role to warn the organism to evade noxious stimuli (such as heat, chemical irritants, and cold) and avoid them in future. However, under injury or disease conditions, pain can persist for months to years, and this type of pain is called pathological or chronic pain. Chronic pain is characterized by spontaneous pain, allodynia (pain evoked by a normally innocuous stimulus), and hyperalgesia (enhanced pain evoked by a noxious stimulus). Changes in neuronal plasticity are the major mechanisms of chronic pain [1]. Thus, several neuron-targeting drugs such as NMDA receptor antagonists, opioids (such as morphine, oxycodone, and codeine), and Na^+^ channel blockers (such as lidocaine, oxcarbazepine, and carbamazepine) are used for the treatment of chronic pain. Although these drugs have therapeutic effects, they also have different degrees of side-effects [2]. Therefore, the development of new types of analgesic with few adverse reactions and better targeting are urgently needed. In the last two decades, non-neuronal cells, especially glial cells, have attracted increasing attention. Targeting the function of glial cells is likely to be a new direction for chronic pain treatment.

In the central nervous system (CNS), more than half of the cells are glia (including astrocytes, microglia, and oligodendrocytes), ~20%–40% of which are astrocytes [3, 4]. Astrocytes not only provide structural and nutritional support for neurons, they also play important roles in many neural processes [5]. Under normal conditions, astrocytes are mostly in a resting state; however, when tissue injury or disease occurs, they transform into a reactive state and contribute to the development of neurological disorders. One of the important features of astrocytes, different from other glial cells, is that they directly communicate with each other by forming gap-junction protein complexes, which allow adjoining cells to freely exchange ions and small cytosolic components [6]. In addition, when CNS neurons are activated, astrocytes regulate blood flow through their extensive contact with cerebral blood vessels [7]. Astrocytes also widely connect with neuronal synapses: a single cortical astrocyte can contact 4–6 neuronal somata, almost 140,000 synapses, and 300–600 neuronal dendrites [3, 4]. Close contact with neurons and synapses makes it possible for astrocytes to support neurons and regulate the physiological/pathological environment during synaptic transmission. These features show that astrocytes play important roles in signal transmission and processing.

In this review, we provide an overview of the roles of astrocytes in the pathogenesis of chronic pain and the interactions between astrocytes and microglia/neurons. We discuss recent neurobiological mechanisms and possible downstream molecular pathways of the astrocytic control of chronic pain. Finally, we discuss how they can be targeted as an alternative strategy for the treatment of chronic pain.

## Spinal Astrocytes in Chronic Pain

Classically, chronic pain is classified into two main categories: nociceptive and neuropathic. Nociceptive pain is associated with an ongoing input from real or threatened tissue injury, such as arthritis, trauma, and visceral inflammation. Neuropathic pain is caused by a direct consequence of a lesion or disease affecting the somatosensory system, such as nerve or nerve root compression, toxins, and ischemia. In 2016, the term nociplastic pain was proposed to describe pain that arises from the abnormal processing of pain signals without any clear evidence of tissue damage or discrete pathology involving the somatosensory system, such as fibromyalgia, irritable bowel syndrome, and temporomandibular disorder [8]. In the past two decades, the role of astrocytes in nociceptive pain and neuropathic pain has been widely studied. Under various disease conditions, astrocytes are activated and change to a reactive state characterized by morphological, molecular, and functional changes. Reactive astrocytes are identified by glial fibrillary acidic protein (GFAP) upregulation and hypertrophy after nerve injuries such as spinal nerve ligation (SNL) [9–12], chronic constriction injury (CCI) [13–15], and spinal cord injury (SCI) [16–18]. In addition, this process has also been reported in other pain models like tissue inflammation [19, 20], chemotherapy-induced pain [21, 22], arthritic pain [23, 24], and chronic post-cast pain [25, 26].

Reactive astrocytes can display various states. Some reactive states happen within minutes, such as a change in the phosphorylation of signaling molecules or an increase in intracellular Ca^2+^. Others occur after hours or days (e.g., astrocyte hypertrophy or translational regulation). When a peripheral nerve is injured, astrocyte hypertrophy occurs 3 days later and lasts for several months [27]. Different from astrocytes, microglia immediately respond to a stimulus and proliferate [28], and this process grows to maximal levels in the first week following nerve injury. Raghavendra *et al.* reported that in a neuropathic pain model, when minocycline (a microglia activation inhibitor) administration is started at the time of nerve transection (pre-emptive treatment), it reduces allodynia and hyperalgesia, which is associated with its ability to suppress microgliosis. However, administration on day 5 after surgery (treatment of existing hypersensitivity) fails to attenuate the behavioral hyperalgesia and allodynia, although it inhibits microglial activation [29]. Meanwhile, astrocyte inhibitors work in both the early and late phases of neuropathic pain [30], indicating that microglia and astrocytes play different roles in the induction and persistence of chronic pain. Evidence shows that microglial reaction may lead to astrocyte reaction [31–34]; however, astrocyte reaction can also cause microglial reaction [33, 35]. A recent study showed that direct activation of astrocytes using an optogenetic approach induces microglial reactivity and pain hypersensitivity [36]. In addition, inhibition of astrocyte reaction by deletion of astrocyte-expressing CXCR5 reduces the microglial reaction under neuropathic pain conditions [37]. These reports indicate that there is a bilateral interaction between microglia and astrocytes in pain states. Thus, we discuss some of the signaling molecules and related signaling pathways in astrocyte-mediated chronic pain, as well as how astrocytes contribute to chronic pain *via* communicating with microglia and neurons.

### Signaling Molecules Related to Astrocyte-mediated Chronic Pain

As noted above, reactive astrocytes not only change in shape, size, and number, but also change in the expression of various molecules. An increasing list of signaling molecules in astrocytes have been implicated in persistent pain, such as cytokines, chemokines, ion channels, enzymes, and structural proteins. Meanwhile, astrocyte reactivity can be triggered by various molecules. Here, we introduce some classical and important signaling molecules in astrocyte-mediated chronic pain (Table 1).

**Table 1:** Astrocyte-selective molecules in chronic pain models

Molecule	Model	Species	Effect of manipulation in the spinal cord	Refs.
*Cytokines, chemokines*				
TNF-α	2′,3′-dideoxycytidine-induced neuropathic pain; SNL; Formalin; CFA	Mouse Rat	Inhibitor or recombinant soluble receptor suppresses mechanical allodynia or thermal hyperalgesia; Inhibitor prevents TNF-*α-*induced phosphorylation of NR1 unit.	44, 45, 50
IL-1β	CFA; SNL	Mouse	Antagonist attenuates inflammatory hyperalgesia; Deletion of related receptor suppresses neuropathic pain.	49, 51, 53, 54
CCL1	pSNL; SNL	Mouse Rat	i.t injection of CCL1 induces phosphorylation of NR1 and NR2B units; Knockdown of related receptor prevents mechanical allodynia and thermal hyperalgesia; Inhibitor of receptor or neutralizing antibody suppresses the development of neuropathic pain.	57, 58
CCL2	SNL; CFA	Mouse Rat	Inhibitor modulates inhibitory synaptic current and suppresses neuropathic pain; CCL2-overexpressing mice show greater edema and hyperalgesia.	62, 63
CXCL10	SNL; ischemia-reperfusion-induced pain	Mouse Rat	Knockdown of related receptor suppresses the development of neuropathic pain; Inhibitor or antagonist of receptor attenuates nociceptive response.	64, 65
*Channel protein*				
AQP4	SCI	Rat	After spinal cord injury; Knockout mice show less pain sensitivity and dorsal horn sensitivity to noxious stimulation.	72, 74
SUR1	PNI	Mouse	Antagonist or knockout suppresses the development of neuropathic pain.	76
P2X3	CCI-ION	Rat	Inhibitor attenuates reactive astrogliosis, release of downstream inflammatory factors, and pain hypersensitivity.	82
*Enzymes*				
MMP-2	SNL	Mouse	Inhibitor suppresses the late phase of neuropathic pain; Knockdown reduces mechanical allodynia and pJNK1/2.	83, 84
MMP-9	SNL	Mouse	Inhibitor suppresses the early phase of neuropathic pain.	83, 84
TAK-1	CCI	Rat	Antisense oligodeoxynucleotide prevents and reverses allodynia but not hyperalgesia and inhibits JNK1 activation.	86, 87
TPA	SNL; SNI	Rat	Inhibitor suppresses mechanical allodynia.	90
*Other molecules*				
NDRG2	Diabetic neuropathic pain	Rat	Blocking its upstream glucocorticoid receptor reverses tactile allodynia.	92
FGFR3	SNI	Mouse	Inhibitor reduces expression of GFAP, TNF-α, and pain hypersensitivity.	95
TRAF6	CFA; Visceral inflammatory pain	Mouse	Knockdown of TRAF6 reduces excitatory postsynaptic currents and inflammatory pain.	96, 98
*Long non-coding RNA*				
PVT1	SCI	Rat	Knockdown down-regulates CXCL13/CXCR5 and alleviates neuropathic pain	99
MEG3	CCI	Rat	Inhibitor weakens MEG3-induced pro-inflammatory effects and relieves pain	101
*Structure-related protein*				
Cx43	CCI; pION	Mouse Rat	Inhibitor suppresses spontaneous excitatory postsynaptic currents and late-phase neuropathic pain; Inhibitor reduces mechanical hypersensitivity and central sensitization.	30, 165
Panx1	CFA	Mouse	Deletion of astrocytic Panx1 prevents hypersensitivity completely; Deletion of neuronal Panx1 reduces baseline sensitivity and duration of hypersensitivity	167

CCI, chronic constriction injury; CCI-ION, constriction injury of infraorbital nerve; CFA, complete Freund’s adjuvant; pION, partial transection of the infraorbital nerve; pSNL, partial sciatic nerve ligation; PNI, peripheral nerve injury; SCI, spinal cord injury; SNI, spared nerve injury; SNL, spinal nerve ligation.

#### Inflammatory Cytokines and Chemokines

Cytokines and chemokines are secreted proteins that regulate the immune responses and control immune cell trafficking. It is well known that cytokines and chemokines in peripheral tissues, dorsal root ganglia (DRG), spinal cord, and even the brain play a certain role in the pathogenesis of chronic pain.

Tumor necrosis factor-α (TNF-α) belongs to a superfamily of ligand/receptor proteins called TNF superfamily proteins. It is a cytokine that is usually produced by activated microglia and astrocytes and has pleiotropic effects on normal and malignant cells [38]. When a peripheral nerve is injured, TNF-α is released from activated microglia and stimulates astrocytes [39–42]. Accordingly, inhibition of TNF-α signaling by neutralizing antibodies or inhibitors alleviates chronic pain [43–45]. Our previous study indicated that spinal injection of TNF-α-activated astrocytes induce persistent pain by releasing CCL2 (C-C Motif Chemokine Ligand 2) [46]. Similar to TNF-α, IL-1β is also expressed in microglia and astrocytes. IL-1β is upregulated in astrocytes in different types of chronic pain such as inflammatory pain [47], neuropathic pain [48], and bone cancer pain [49]. IL-1β activates the IL-1 receptor, which is expressed on nociceptive neurons and activates the mitogen-activated protein kinase (MAPK) pathway and sensitizes the neurons. IL-1β and TNF-α regulate the phosphorylation of the NR2B and NR1 subunit of the NMDA (N-methyl D-aspartate) receptor [50] and enhance NMDA-induced currents [51, 52], which suggest a potentiation of glutamatergic synaptic transmission. TNF-α and IL-1β also increase the frequency and amplitude of spontaneous excitatory postsynaptic currents (sEPSCs) [53]. IL-1 receptor-knockout mice show decreased nociceptive responses after SNL [54]. In addition, optogenetic activation of astrocytes causes an increase of IL-1β and TNF-α secretion [36]. In human studies, TNF-α and IL-1β are also increased in the spinal astrocytes of patients with HIV-associated chronic pain [55]. These results suggest the important role of TNF-α and IL-1β in regulating neuropathic pain.

In addition to cytokines, several chemokines such as CCL1, CCL2, CCL3, CCL4, CCL7, CXCL10, CXCL12, CXCL13, and CX3CL1 and their receptors contribute to the pathogenesis of neuropathic pain [56]. Here, we focus on the chemokines or chemokine receptors associated with astrocytes. CCL1 was initially identified in T cells and stimulates the migration of human monocytes through binding to its receptor CCR8. In the SNL model, CCL1 is mainly produced in the DRG and transported to the spinal cord [57]. Meanwhile, CCR8 is increased in astrocytes of the ipsilateral superficial dorsal horn. Inhibition of CCL1 by intrathecal injection of a neutralizing antibody reduces nerve ligation-induced tactile allodynia [57]. Also, oral administration of RAP-103, a peptide inhibitor of CCR8, fully prevents mechanical allodynia and inhibits the development of thermal hyperalgesia after SNL, suggesting the involvement of CCR8 in the initiation and maintenance of nerve injury-induced neuropathic pain [58, 59].

CCL2 is highly expressed by spinal astrocytes and is upregulated in the SNL model [46]. Meanwhile, CCL2 is produced in cultured astrocytes after stimulation with lipopolysaccharide (LPS), TNF-α, or IL-1β [60]. CCR2 is the major receptor of CCL2 and is expressed in primary afferents and neurons in the spinal cord [61]. Mice overexpressing CCL2 in astrocytes display enhanced nociceptive responses in the CFA (complete Freund’s adjuvant) model [62]. Our previous study demonstrated that CCL2 induces rapid phosphorylation of ERK (extracellular signal-activated kinase) in spinal cord neurons. In addition, when lamina II neurons in the spinal cord slice are recorded, the application of CCL2 immediately enhances NMDA- and AMPA-induced inward currents and causes an increase in the frequency and amplitude of sEPSCs [46]. CCL2 also modulates inhibitory synaptic transmission since it inhibits GABA-induced currents in spinal neurons [63].

CXCL10 belongs to the CXC chemokine family and is also known as keratinocyte-derived chemokine-10. CXCL10 is the major ligand of CXCR3 and is increased in neurons and astrocytes of the spinal cord after SNL or spinal cord ischemia reperfusion [64, 65]. Inhibition of CXCL10 by spinal injection of shRNA lentivirus attenuates SNL-induced mechanical allodynia and heat hyperalgesia [64, 66]. CXCL9 and CXCL11 belong to the same subfamily as CXCL10 [67]. However, the roles of these chemokines in pain hypersensitivity are different from CXCL10. Intrathecal injection of CXCL9 or CXCL11 does not induce hyperalgesia or allodynia behaviors, and their inhibition does not inhibit neuropathic pain either [68], suggesting different roles of these chemokines in pain regulation. Other chemokines and their receptors such as CX3CL1/CX3CR1, CXCL1/CXCR2, and CXCL12/CXCR4 are also involved in chronic pain and have been introduced in our previous and others’ reviews [56, 69, 70], so they are not discussed in detail here.

#### Channel Proteins

Different types of cationic or anionic channels are located on astrocytic membranes to regulate ions for the resting membrane potential or conductance and intracellular signaling. The ion channels on astrocytes are also involved in regulating the release of various gliotransmitters associated with several physiological processes. Here, we introduce some typical water and ion channels that are expressed on astrocytes involved in chronic pain regulation.

Aquaporin-4 (AQP4) is a major water channel expressed in the central nervous system, primarily in astrocytes. The role of AQP4 has been widely studied in a range of pathological conditions [71]. In the spinal cord, AQP4 exhibits a graded decline in distribution from the dorsal to the ventral horn, with abundant expression in laminae I and II [72]. The function of AQP4 is to regulate water influx or efflux driven by osmotic pressure to maintain water homeostasis. AQP4 is increased in spinal cord astrocytes after SCI and nerve injury in humans [73]. In addition, AQP4-knock-out mice show reduced pain sensitivity and dorsal horn sensitivity to noxious stimulation [74].

Sulfonylurea receptor 1 (SUR1), encoded by the *Abcc8* gene, is a regulatory subunit that co-assembles with the inward rectifier K^+^-selective channel to form the K_ATP_ channel [75]. SUR1 also co-assembles with the non-selective cation channel, transient receptor potential melastatin 4 (TRPM4) to form the SUR1-TRPM4 complex. SUR1-TRPM4 is upregulated in dorsal horn astrocytes, and global or astrocytes-targeted deletion of SUR1-TRPM4 relieves mechanical allodynia and thermal hyperalgesia in a sciatic nerve cuffing mouse model [76]. Meanwhile, chronic administration of glibenclamide (an SUR1 antagonist) to mice with neuropathic pain causes a reduction of pain behaviors and the expression of IL-6, CCL2, and CXCL1 in astrocytes. Thus, glibenclamide may be an astrocyte-targeted candidate drug for the treatment of some kinds of neuropathic pain.

P2X3 is a non-selective ligand-gated ion channel that belongs to the purinergic receptor family [77]. P2X3 is activated by adenosine triphosphate (ATP) and is selectively permeable to Na^+^, K^+^, and Ca^2+^, especially Ca^2+^, which plays an important role in the generation and transmission of nociceptive information [78]. Nerve injury causes the release of a large amount of ATP, which activates the P2X3 receptor in the presynaptic membrane and causes Ca^2+^ influx, resulting in phosphorylation of PKA and PKC and the release of glutamate. This process further activates the NMDA receptors on neurons and causes EPSC generation and central sensitization [79]. It is well known that activation of P2X3 in the DRG causes abnormal nerve discharge, strengthens the transmission of sensory information, and induces visceral hyperalgesia [80, 81]. P2X3 is also expressed on astrocytes in the spinal cord and is increased in a rat model of neuropathic pain [82]. Inhibition of P2X3 in the spinal cord reduces hypersensitivity after nerve injury. In addition, administration of MPEP (2-methyl-6-(phenylethynyl)pyridine; an mGluR5 antagonist) reduces the mechanical allodynia and abolishes the increase in the density of P2X3 in astrocytes induced by nerve injury [82].

#### Enzymes

Metalloproteases (MMPs) have been suggested to act in the cleavage of extracellular matrix proteins, cytokines, and chemokines to control the inflammation and tissue remodeling associated with various neurodegenerative diseases [83]. MMP-2 and MMP-9 are members of the MMP family involved in IL-1β cleavage [83, 84]. Spinal astrocytes continuously secrete MMP2 after SNL. Downregulation of MMP-2 through intrathecal injection of MMP-2 siRNA reduces mechanical allodynia and the level of spinal GFAP and phosphorylated c-Jun N-terminal kinase 1/2 (JNK1/2), an astrocyte-expressing kinase, in a neuropathic pain model. Local inhibition of MMP-9 inhibits the early phase of neuropathic pain, whereas inhibition of MMP-2 suppresses the late phase of neuropathic pain [83].

Transforming growth factor-β-activated kinase 1 (TAK-1), also known as MAPK kinase kinase 7, is an enzyme regulating innate immunity and pro-inflammatory signaling [85]. TAK-1 mediates the activation of the nuclear factor-kB (NF-kB), JNK, and p38 pathways. Soto-Diaz *et al.* found that both the production of chemokines and neutrophil migration caused by astrocyte reaction are dependent on TAK1 signaling [86]. Another study reported by Katura *et al.* showed that peripheral nerve injury induces an increase in TAK1 expression in astrocytes in the spinal dorsal horn, and this TAK1 upregulation increases JNK1 phosphorylation in spinal astrocytes and contributes to the development and maintenance of mechanical hypersensitivity [87].

Tissue type plasminogen activator (tPA) is a well-known extracellular serine protease that converts zymogen plasminogen into an active serine protease. tPA is found on the endothelial cells of blood vessels and is involved in the degradation of blood clots [88]. In addition, tPA participates in modification of the extracellular matrix that leads to long-term potentiation in the hippocampus [89]. Kozai *et al.* reported that tPA is upregulated in spinal astrocytes following root injury [90]. Moreover, continuous intrathecal administration of a tPA inhibitor suppresses root ligation-induced mechanical allodynia. These data suggest that astrocyte-derived tPA in the dorsal horn is essential for the mechanical hypersensitivity following root injury.

#### Other Molecules

N-myc downstream-regulated gene 2 (NDRG2) is a member of the NDRG family and is widely distributed in the CNS but only expressed in astrocytes. NDRG2 members have different functions in cell differentiation, proliferation, and maintenance of cell morphology [91]. Ma *et al.* found that down-regulation of NDRG2 in spinal astrocytes inhibits their reactivity and reduces nociceptive behaviors in a rat model of spared nerve injury (SNI) [92]. Another study reported by Li *et al.* also indicated that inhibition of NDRG2 contributes to astrocyte-specific neuroprotection [93].

FGFR3 is a member of the fibroblast growth factor receptor (FGFR) family, which contains four members (FGFR1–4) that mediate FGF signal transduction. FGF/FGFR signaling plays an important role in cell differentiation, neuronal survival, and cell development [94]. Previous studies have shown that activated FGFR3 promotes the proliferation and development of astrocytes [95]. Xie *et al.* showed that FGFR3 upregulates GFAP and TNF-α expression in astrocytes *in vivo* and *in vitro*. Inhibition of FGFR3 leads to reduced GFAP and TNF-α and increases the withdrawal threshold in SNI model [95]. These results suggest that FGFR3 induces production of the inflammatory mediator TNF-α and astrocyte reactivity to cause hyperpathia in neuropathic pain.

TRAF6 belongs to the TNF receptor-associated factor (TRAF) protein family which has 6 members (TRAF1–TRAF6). A growing body of literature has established the important role of TRAF6 in the development and maintenance of chronic pain. Our previous study showed that TNF-α, IL-1β, and TLR4 mediate TRAF6 upregulation in spinal astrocytes after SNL in mice [96]. However, TRAF6 is increased in spinal microglia in CFA-induced chronic inflammatory pain [97]. Direct inhibition of TRAF6 by siRNA or indirect inhibition by docosahexaenoic acid has therapeutic effects on neuropathic pain and inflammatory pain [97]. Another study showed that TRAF6 is increased in spinal astrocytes in the chronic visceral pain model [98]. Knockdown of TRAF6 remarkably reduces the amplitude of EPSCs of spinal dorsal horn neurons and relieves visceral hypersensitivity [98].

Long non-coding RNAs (lncRNAs) have hundreds of nucleotides with no protein-coding potential. Currently, there is growing evidence that lncRNAs play an important role in regulating chronic pain. For example, lncRNA PVT1 is up-regulated in spinal astrocytes after SCI [99]. Depletion of PVT1 in the spinal cord reduces nociceptive responses such as thermal hyperalgesia and mechanical allodia as well as the expression of neuroinflammatory factors and proteins. Furthermore, it has been confirmed that PVT1 is a competitive endogenous RNA of miR-186-5p, while miR-186-5p targets CXCL13 [99]. Inhibition of lncRNA PVT1 alleviates neuropathic pain in SCI rats by upregulating miR-186-5p and down-regulating CXCL13/CXCR5 [99, 100]. Another similar study showed that lncRNA MEG3 aggravates neuropathic pain and astrocyte reactivity by mediating the miR-130a-5p/CXCL12/CXCR4 axis [101].

## Interaction of Astrocytes, Microglia, and Neurons Under Chronic Pain Conditions

It is well-accepted that neuronal plasticity is a key mechanism for the development and maintenance of chronic pain. Astrocytes and microglia are important players in the regulation of neuronal functions [102]. The communication between astrocytes, microglia, and neurons in the spinal cord and brain facilitates central sensitization, which is manifested as an increased responsiveness of neurons to normal or subthreshold afferent inputs.

Microglia are the resident immune cells of the CNS. They are activated by tissue damage or nerve injury [28] and their morphology changes to an amoeboid shape, accompanied by enhanced secretion of numerous inflammatory factors and microglial phagocytosis [3]. Evidence has already demonstrated that microglia play an important role in the pathogenesis of chronic pain [28]. With different stimuli (usually extracellular/intracellular signals like inflammatory mediators or anti-inflammatory mediators), microglia display two phenotypes, M1 and M2 [33]. The M1-like phenotype is induced by TNF-α, IL-1β, or other inflammatory mediators, and then microglia secrete pro-inflammatory cytokines such as IL-6, IL-23, or chemokines (CCL2 and CCL5) to induce neuroinflammation, resulting in the maintenance of chronic pain [28]. The M2-like phenotype is induced by IL-4 or IL-13. Microglia with M2-like phenotypes have increased phagocytosis and produce growth factors such as insulin-like growth factor-1 and anti-inflammatory cytokines such as IL-10 [103]. The cross-talk between astrocytes and microglia is maintained in part *via* secreted mediators, such as growth factors, neurotransmitters, cytokines, chemokines, innate-immunity mediators, tissue damage molecules (e.g., ATP), mitogenic factors, NO, ROS, and metabolic mediators such as amino-acids, that can be used for cell metabolism and may also mediate tissue changes [104]. The production and release of these mediators are normally controlled by key intracellular signaling pathways, such as the MAPK pathway. Many chemokines, such as CXCL12, CXCL10, CXCL1, and CCL2 are mainly stored in astrocytes, and microglia express the corresponding chemokine receptors like CXCR4 or CCR2 [105]. This suggests a strong association between microglia and astrocytes [6]. IL-18, one member of the IL-1 family, is an important regulator of innate and acquired immune responses. Kan *et al.* reported that IL-18 and IL-18 receptors are expressed in microglia and astrocytes, respectively, mediate microglia-astrocyte interaction in the spinal cord, and enhance neuropathic pain processing after nerve injury [106]. These results suggest that activated microglia in the spinal dorsal horn are directly responsible for the induction of astrocyte reactivity after nerve injury. However, reactive astrocytes sometimes are not correlated with reactive microglia. Hald *et al.* found that bone cancer markedly induces spinal astrocyte reactivity, not microglial reactivity [107]. In addition, Robinson *et al.* reported that astrocyte but not microglial reactivity is induced in oxaliplatin- and bortezomib-induced peripheral neuropathy in rats [108].

In addition to the interaction with microglia, astrocytes also directly interact with neurons. We found that CXCL13-CXCR5 mediates neuron-astrocyte interaction in the spinal cord after SNL [37]. SNL increases the expression of CXCL13 and its receptor CXCR5 in neurons and astrocytes in mice. In *Cxcr5*-KO mice, the reactivity of astrocytes and microglia in the dorsal horn is remarkably reduced after nerve injury [37]. Furthermore, when astrocytes are activated, they release CCL2 and CXCL1 to act on their receptors CCR2 and CXCR2 on spinal neurons, thus causing an enhancement of excitatory synaptic transmission and chronic pain [46, 56, 109, 110]. Recent work has shown that CXCL2 secreted by astrocytes interacts with CXCR2 expressed on neurons in the spinal cord and this contributes to carrageenan-induced prostatitis pain [111]. Meanwhile, the CXCL1/CXCR2-mediated interaction between astrocytes and neurons in the periaqueductal gray (PAG) also facilitates chronic pain [112]. A recent study showed that Wnt5a from neurons is crucial for reactive astrogliosis in an animal model of HIV-associated pain [113]. Wnt5a from neurons targets its receptor ROR2, which is expressed in astrocytes. Furthermore, conditional knockout of either Wnt5a in neurons or its receptor ROR2 abolishes not only gp120-induced astrocyte reactivity but also hyperalgesia. These results show that astrocytes, microglia, and neurons form a loop to interact with each other and regulate chronic pain.

## The Intracellular Signaling Pathway in Reactive Astrocytes in Chronic Pain

Several intricate roles are played by astrocytes in the pathogenesis of chronic pain, not only in the means of intercellular communication but also in the alteration of intracellular downstream signaling pathways as well as the variation in metabolic patterns. Nerve injury or pathogen invasion can be the initial factor for astrocyte activation in chronic pain. Here, we introduce astrocytic intracellular signaling and the actions of astrocyte-released neuromodulators in chronic pain. Some typical pathways such as the p38 and JNK pathways have been described in previous reviews [4, 6], thus we do not discuss them in detail.

Janus kinase (JAK) signal transducers and activators of the transcription 3 (STAT3) signaling pathway is involved in the restricted proliferation of dorsal horn astrocytes after peripheral nerve injury [114]. Inhibition of JAK-STAT3 suppresses both the proliferation of dorsal horn astrocytes and the maintenance of tactile allodynia. It has been shown that the dimerization of gp130 participates in the conduction pathway of activated JAK1 and JAK2, followed by phosphorylating STAT3 [115]. Gp130 cooperates with other receptors such as IL-6, IL-11, and IL-27 to constitute receptor complexes [116–118]. Considering the pleiotropic effects and widespread expression of these cytokines, the gp130-JAK-STAT3 signaling pathway may provide new directions in the research on reactive astrogliosis concerned with chronic pain.

NF-κB plays important roles in many aspects of cell regulation such as proliferation, apoptosis, and differentiation [119]. NF-κB can regulate neuropathic pain by mediating neuroinflammation, neuron apoptosis, and synaptic plasticity [120]. NF-κB is also strongly activated in reactive astrocytes and contributes to the development of neuropathic pain. During the progression of SCI, the inactivation of NF-κB in transgenic GFAP-IκBα-dn mice causes a reduction of lesion volume, an increase of white matter preservation, and a reduction in the expression of proteoglycan and chemokines CXCL10 and CCL2, and improves functional recovery [121]. During the inflammatory pain process, NF-κB plays an important role in gene transcription induced by cytokines. IL-1β and TNF-α activate NF-κB and lead to the transcription of several genes including pro-inflammatory factors and chemokines. This is a positive feedback process that further activates NF-κB and leads to more expression of downstream genes associated with neuroinflammation. In addition, Toll-like receptors (TLRs) are key regulators of the NF-κB signaling pathway. TLR4 regulates neuropathic pain through its activation of microglia and astrocytes in the spinal cord. When TLR4 is activated by LPS or other stimuli, NF-κB acting as its main downstream pathway is activated to begin the next process to develop chronic pain [122]. Using different methods including blockers or siRNA to inhibit the NF-κB signaling pathway can relieve different types of chronic pain. Thus, regulation of the NF-κB signaling pathway could be a potential therapeutic strategy [121, 123, 124].

The transcription factor Olig2 is considered to play an essential role in the differentiation of oligodendrocytes and motor neurons in the embryonic spinal cord. However, recent findings suggest that Olig2-lineage astrocytes are a different subgroup from GFAP-lineage astrocytes, both of which frequently occupy mutually exclusive territories and have distinct mRNA expression patterns [125–127]. In addition, Olig2-lineage astrocytes tend to express GABA transporter-3 and are involved in inhibitory neuronal transmission [126]. Ablation of Olig2 decreases the proliferation of reactive astrocytes in response to injury [128]. Olig2 is a direct target of Notch signaling [129], which upregulates Olig2 expression and promotes Olig2 localization in the nucleus in reactive astrogliogenesis in the brain. Inhibitor of the Notch-activating enzyme reduces the number of reactive astrocytes [130]. Given the fact that Olig2 influences Wnt signaling in gliomas and neural stem cells and participates in the interaction between Wnt signaling and Notch signaling, further research on the Notch-Olig2-Wnt pathway is important in the area of astrogliogenesis and chronic pain [131, 132].

TGF-β is rapidly and chronically elevated in response to CNS injury. Intrathecal injection of TGF-β inhibits neuropathy-induced hyperalgesia as well as spinal microglial and astrocytic reactivity [133, 134]. TGF-β attenuates the upregulation of pp38 and pERK in spinal microglia and astrocytes of mice with CCI. Despite the neuroprotective role of TGF-β, it also stimulates astrocytes to a reactive state with up-regulated GFAP [135]. Schachtrup *et al.* emphasized that fibrinogen is a carrier of latent TGF-β and induces phosphorylation of Smad2 in astrocytes after leakage through the disrupted blood–brain barrier or vascular damage [136]. Nuclear Smads interact with a large number of transcription factors to activate target genes [137]. TGF-β regulates adenine nucleotide translocator 1 gene expression, which is responsible for the removal of extracellular glutamate through a cooperative interaction of both Smad and Sp1 binding elements located immediately upstream of the transcriptional start site [138, 139].

## Supraspinal Astrocytes in Chronic Pain

Chronic pain results from peripheral and central sensitization [1]. Central sensitization occurs not only in the spinal cord but also in supraspinal areas. Noxious stimuli are detected and transduced into electrical signals and further transmitted from the DRG to the dorsal horn. The signal is then sent up through the spinothalamic tract to the thalamus and then to the primary somatosensory cortex (S1) [140]. Several areas such as the PAG, parabrachial nucleus, nucleus accumbens, and anterior cingulate cortex (ACC) are important for pain regulation [141]. Under nerve injury or inflammatory conditions, astrocytes undergo varied changes in the brainstem, thalamus, ACC, and S1 [142, 143], and these changes contribute to central sensitization and chronic pain.

The spinal trigeminal nucleus is similar to the spinal dorsal horn and plays an essential role in trigeminal pain transmission. Reactive astrocytes in the spinal trigeminal nucleus contribute to the pathogenesis of chronic orofacial pain [144]. In reactive astrocytes, the TNF signaling pathway is activated and increases the expression of GFAP, CX43, and IL-1β, leading to neuropathic pain in the trigeminal system [48, 145]. This activation process of astrocytes is initiated by upregulation of several marker genes, including complement 3, complement factor B, MX dynamin-like GTPase 1, and S100a10 [32]. Neurons and glial cells regulate this astrogliosis process through various signaling pathways, including the JAK-STAT3, Notch-OLIG2, and TGFβ-SMAD pathways [114, 128, 146–148]. In addition, after infraorbital nerve injury, reactive astrocytes are detected in the rostral ventromedial medulla (RVM), a major component of the brainstem for descending pain modulatory circuitry [48]. The expression levels of the pro-inflammatory factors TNF-α and IL-1β are increased in RVM astrocytes. Intra-RVM administration of astrocytic inhibitors attenuates mechanical hyperalgesia and allodynia behaviors. Moreover, TNFR1 and IL-1R are expressed in RVM neurons that express the NMDA receptor subunit NR1. Injection of recombinant TNF-α or IL-1β upregulates NR1 phosphorylation and causes an NMDAR-dependent allodynia [48]. Supraspinal astrocytes also participate in descending nociceptive modulation in a cancer-induced bone pain model. Ni *et al.* showed that activation of astrocytes in the ventrolateral PAG is implicated in facilitating bone cancer pain in rats *via* the JNK signaling pathway [149]. Intrathecal administration of astrocytic cytotoxin or a JNK inhibitor reduces the expression of GFAP and mechanical allodynia. All the above suggest a contribution of supraspinal astrocytes and central glia-neuronal interactions to the descending facilitation of chronic pain.

Astrocytes in other regions of the brain have also been reported to be involved in the pain matrix such as the ACC and S1. Astrocytes in the ACC play a role in the affective component of pain, including unpleasantness or aversion [142]. SNL activates astrocytes in S1 and increases mGluR5 expression [143]. This leads to an increase in S1 astrocytic Ca^2+^ transients and, thus, the release of thrombospondin 1 from astrocytes, which promotes synapse formation as well as mechanical allodynia [143]. In addition, Wahis *et al.* reported a new function of astrocytes in the central nucleus of the amygdala (CeA) under neuropathic pain conditions [150]. They found that activation of oxytocin receptors (OTRs) in the CeA reduces neuropathic pain-induced anxiety behavior. Meanwhile, the deletion of OTRs from the astrocytes in the lateral part of the CeA abolishes the anxiolytic effects of OTRs agonists [150]. These data highlight the central role of astrocyte-mediated oxytocin signaling in the regulation of emotional states under chronic pain conditions.

It is important to note that the research above was mostly based on animal studies. Human astrocytes have several distinct properties that are quite different from those of rodents. The human brain contains subtypes of GFAP-positive astrocytes that are not expressed in rodents [135]. Also, the size of human cortical astrocytes is almost double that in rodents [151]. Real-time imagining studies on the brains of patients with chronic lower back pain have demonstrated glial activation in multiple regions including the thalamus and S1 [152]. This research suggests possible astrocyte activation in higher brain regions in humans. Thus, determining whether there is concomitant neuronal activation with astrocytes activation in chronic pain may be of major importance in future research.

## Targeting Astrocytes as an Alternative Strategy for the Treatment of Chronic Pain

Long-term unendurable and severe chronic pain impairs patients’ quality of life and imposes a heavy economic burden on society. Non-steroidal anti-inflammatory drugs, opioids, and adjuvant analgesics like antidepressants and antiepileptics are common pharmacological treatments for managing chronic pain. However, most patients face the problem of receiving inadequate analgesic therapy and the prescription is limited by the side-effects including gastrointestinal hemorrhage, thrombotic cardiovascular events, and addiction [153]. Hence, there is an urgent need for developing new therapeutic strategies for chronic pain.

With the improving understanding of the roles of astrocytes in chronic pain, therapeutic strategies may target their underlying mechanisms in order to reduce pain or enhance the recovery process. For example, matrix metalloproteinases (MMPs), particularly MMP-2, are known to participate in neuropathic pain after nerve injury [83, 84]. MMP-2 is released by astrocytes after the injury and induces activation of IL-1β [83]. Therefore, it would be ideal to target MMP-2 and reduce inflammation after injury. Unfortunately, most available drugs have a non-specific affinity for MMPs and thus induce various side-effects or minimal desired effects. MMP-1 and MMP-9 inhibitors have recently been developed, and yet not much progress has been made in the development of MMP inhibitors [154]. TNF-α is a cytokine that is usually produced by activated microglia and astrocytes. Administration of TNF-α antibody effectively alleviates hyperalgesia [155], indicating the prospect of anti-TNFα therapy in the treatment of chronic pain. An ongoing phase III clinical trial is aimed to test the efficacy of infliximab (a TNFα antagonist) in treating lower-back pain in patients (NCT03704363). Multiple compounds and medicines targeting astrocytes alleviate chronic pain by regulating pro-inflammatory mediators such as TNFα, IL-1β, and CCL2 [156–160]. Of note, besides the neuronal expression of TNFR1 and IL-1R, they are also expressed in astrocytes and microglia and contribute to glial activation [45, 96, 161]. Pharmacological inhibition of TNF-α also attenuates glial activation then relieves chronic pain. However, since a certain amount of TNFR1 or IL-1R is expressed on neurons, blocking TNF-α also affects neuronal function.

In addition, pharmacological inhibition of P2X3 in rats following CCI of the trigeminal infraorbital nerve attenuates facial pain [82]. The purinergic receptor P2X3 is found in astrocytes in the spinal trigeminal nucleus, and blocking P2X3 inhibits reactive astrogliosis and the release of downstream inflammatory factors [82]. Thus, it might also be possible to target downstream molecules of reactive astrogliosis to reduce its effect on pain behaviors. For example, patients with neuromyelitis optica have been treated with tocilizumab, an IL-6 antibody, which was found to be safe and effective [162]. Although some of these compounds have antinociceptive effects in animal models and inhibit the reactivity of astrocytes, more designs for clinical trials to test their analgesic efficacy on humans are needed.

Another potential approach is to block gap junction proteins, such as connexin-43 (Cx43) and pannexin 1 (Panx1). Cx43 is specifically upregulated in spinal astrocytes after CCI [30]. Once upregulated, Cx43 enhances ATP release from astrocytes and finally leads to microglial activation and allodynia [163, 164]. Besides, Cx43 also contributes to the release of glutamate and chemokines, and blocking this protein remarkably attenuates neuropathic pain sensitization [30, 165]. The main mechanism is that, in chronic pain states, Cx43 modulates hemichannel function, leading to an increase in the permeability to various cytokines and chemokines [30, 166]. In addition, glial Panx1 contributes to the tactile hypersensitivity in chronic orofacial pain by inducing hyper-responsiveness to ATP [167]. Some Panx1 blockers (including mefloquine and probenecid) have been reported to improve morphine withdrawal syndrome [168]. However, further studies are needed to clarify their effects on humans.

## Conclusion and Future Perspectives

**Fig. 1:**
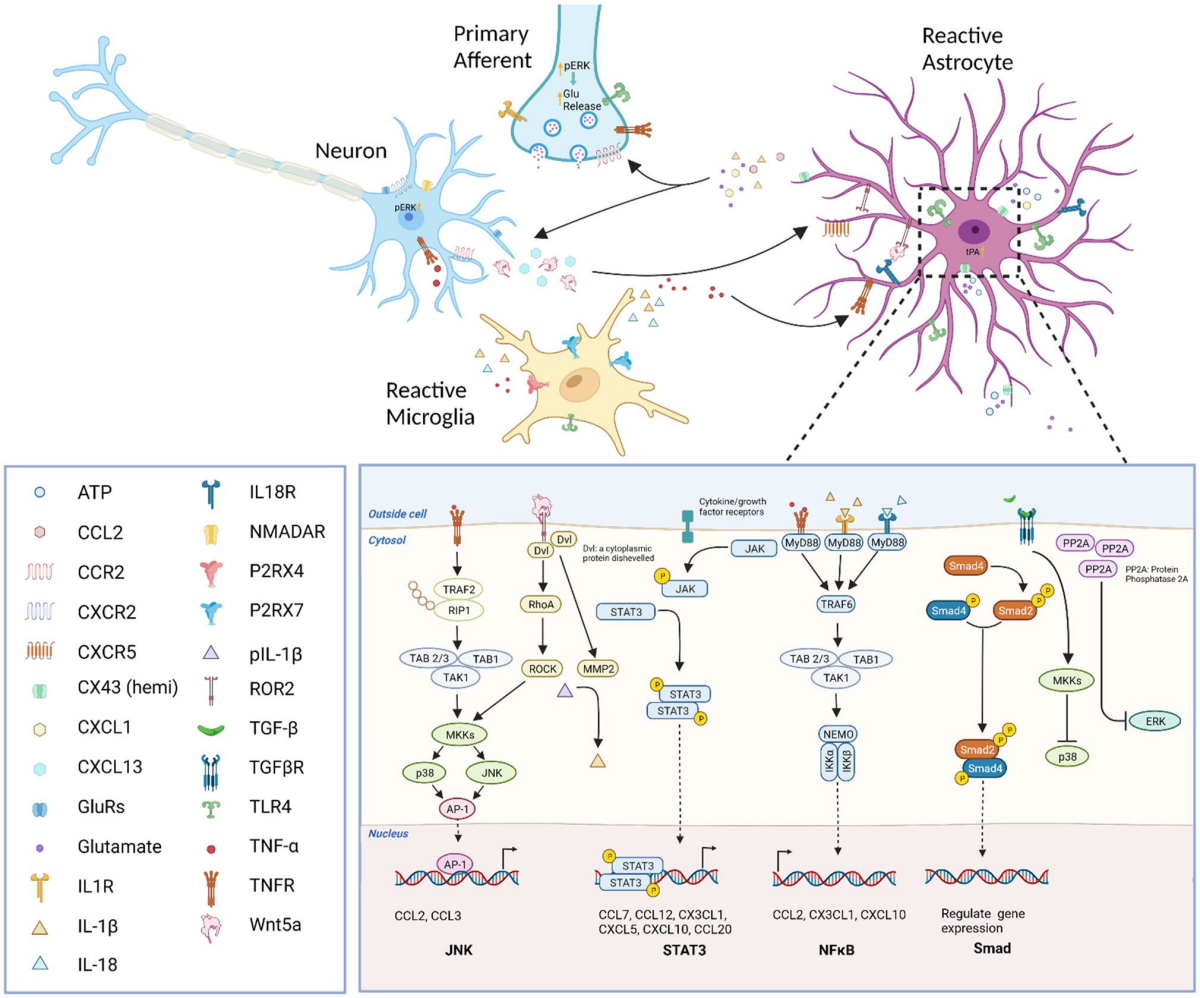
Astrocytic, microglial, and neuronal interaction in chronic pain. Nerve injury induces the release of CXCL13, which activates astrocytes *via* the CXCR5 receptor. The activation of astrocytes results in the upregulation of CX43 expression and a switch in CX43-mediated function from gap-junction communication to CX43 hemichannel-mediated paracrine signaling, resulting in the increased release of pro-inflammatory cytokines, chemokines, glutamate, and ATP, which activate microglia through P2RX4, P2RX7, and other receptors. The activation of these microglial receptors induces the release of pro-inflammatory cytokines (including TNF-α and IL-1β) and further amplifies neuronal excitability. These cytokines also result in the upregulation of the transcriptional regulators TRAF6, STAT3, and subsequent activation of the JNK and ERK pathways in astrocytes, further increasing their production and release of chemokines and facilitating neuropathic pain. The figure was created with BioRender.com.

In summary, we have reviewed different kinds of evidence to demonstrate the necessity and sufficiency of astrocytes in chronic pain. We also explain how astrocytes promote chronic pain through astrocyte-microglia or astrocyte-neuron interactions (Fig. 1). When peripheral nerve injury or tissue damage occurs, astrocytes change to a reactive state in response to different neurotransmitters or neuromodulators in the spinal cord or brain. Reactive astrocytes are usually accompanied by the activation of a variety of intracellular signaling pathways. Therefore, the molecular mechanisms of astrocyte-microglia-neuron crosstalk in the spinal cord and brain under chronic pain conditions deserve further study.

Given the important role of astrocytes in the facilitation of chronic pain, targeting them may provide novel prevention and treatment strategies. However, because astrocytes play an essential supportive and protective role in the CNS, it is important to target specific signaling events in astrocytes without disrupting their overall well-being. The recent identification of astrocyte-expressing genes by transcriptome analyses suggests that astrocytes display inter- or intra-regional heterogeneity and act as a gate for descending noradrenergic control of mechanosensory behavior, which indicates the diverse functions and phenotypes of astrocytes for chronic pain regulation [169]. Akdemir *et al.* also found that subpopulations of Lfng-labeled astrocytes in laminae III and IV of the dorsal horn are involved in the regulation of neuronal activity and maintaining sensory-processing circuity associated with light touch [170]. Ablation of Lfng+ astrocytes reduces glutamatergic synapses and mechanosensory responses. In addition, compared to the classical neuronal gate control theory of pain, Xu *et al.* reported a new function of astrocytes in the gating of nociceptive signals in the spinal cord [171]. Spinal astrocytes are activated by electrical stimulation of peripheral Aβ fibers, which induces long-term depression in NK1R+ neurons and antinociception. Meanwhile, suppression of reactive astrocytes by different methods blocks such processes. Their results demonstrate astrocytes as a new and important component of pain gating by activation of Aβ fibers that exert non-neuronal control of pain. Thus, these recent discoveries may provide a new research direction for astrocyte regulation of chronic pain.
